# NNAT is a novel mediator of oxidative stress that suppresses ER + breast cancer

**DOI:** 10.1186/s10020-023-00673-y

**Published:** 2023-07-03

**Authors:** Cody Plasterer, Marharyta Semenikhina, Shirng-Wern Tsaih, Michael J Flister, Oleg Palygin

**Affiliations:** 1 Department of Physiology, Medical College of Wisconsin, Milwaukee, WI USA; 2 Cancer Center, Medical College of Wisconsin, Milwaukee, WI USA; 3 Genomic Sciences and Precision Medicine Center (GSPMC), Medical College of Wisconsin, Milwaukee, WI USA; 4 Division of Nephrology, Department of Medicine, Medical University of South Carolina, Charleston, South Carolina USA; 5 Department of Regenerative Medicine and Cell Biology, Medical University of South Carolina, Charleston, South Carolina USA

**Keywords:** Breast cancer, Neuronatin, PPAR, ROS, Orai

## Abstract

**Background:**

Neuronatin (NNAT) was recently identified as a novel mediator of estrogen receptor-positive (ER+) breast cancer cell proliferation and migration, which correlated with decreased tumorigenic potential and prolonged patient survival. However, despite these observations, the molecular and pathophysiological role(s) of NNAT in ER + breast cancer remains unclear. Based on high protein homology with phospholamban, we hypothesized that NNAT mediates the homeostasis of intracellular calcium [Ca^2+^]_i_ levels and endoplasmic reticulum (EndoR) function, which is frequently disrupted in ER + breast cancer and other malignancies.

**Methods:**

To evaluate the role of NNAT on [Ca^2+^]_i_ homeostasis, we used a combination of bioinformatics, gene expression and promoter activity assays, CRISPR gene manipulation, pharmacological tools and confocal imaging to characterize the association between ROS, NNAT and calcium signaling.

**Results:**

Our data indicate that NNAT localizes predominantly to EndoR and lysosome, and genetic manipulation of NNAT levels demonstrated that NNAT modulates [Ca^2+^]_i_ influx and maintains Ca^2+^ homeostasis. Pharmacological inhibition of calcium channels revealed that NNAT regulates [Ca^2+^]_i_ levels in breast cancer cells through the interaction with ORAI but not the TRPC signaling cascade. Furthermore, NNAT is transcriptionally regulated by NRF1, PPARα, and PPARγ and is strongly upregulated by oxidative stress via the ROS and PPAR signaling cascades.

**Conclusion:**

Collectively, these data suggest that NNAT expression is mediated by oxidative stress and acts as a regulator of Ca^2+^ homeostasis to impact ER + breast cancer proliferation, thus providing a molecular link between the longstanding observation that is accumulating ROS and altered Ca^2+^ signaling are key oncogenic drivers of cancer.

**Supplementary Information:**

The online version contains supplementary material available at 10.1186/s10020-023-00673-y.

## Background

Metabolic reprogramming that coincides with oxidative stress and remodeling of intracellular calcium (Ca^2+^) signaling are hallmarks of cancer [1]. Under normal physiological conditions, free intracellular levels of Ca^2+^ are tightly maintained at ~ 100-150nM until Ca^2+^ is released downgradient from intracellular stores (e.g., endoplasmic reticulum (EndoR), Golgi, or lysosome) or across the plasma membrane to reach intracellular levels of > 1 μm [2]. Upon triggered release by a variety of stimuli, Ca^2+^ acts as a second messenger to diverse physiological pathways ranging from cell growth and motility to apoptotic cell death, depending on cell state and context [3]. Due to the rewiring of intracellular Ca^2+^ handling pathways, tumor cells may inhibit or otherwise adjust their intracellular Ca^2+^ signaling and concomitant increase in reactive oxygen species (ROS) [4], which contribute to immortalized growth, motility, and survival under conditions that would otherwise trigger cell senescence or death [2]. However, despite the mounting evidence that Ca^2+^ and ROS are critical hallmarks of cancer, the underlying molecular mechanisms that tumor cells exploit to rewire Ca^2+^ and ROS signaling for disease pathogenesis remain largely unknown.

The EndoR is a major store of intracellular Ca^2+^ that is maintained by the activity of sarco/endoplasmic reticulum Ca^2+^-ATPase (SERCA2) to transport Ca^2+^ from the cytoplasm into the EndoR [5]. SERCA2 activity is regulated by phospholamban (PLN) binding to maintain the EndoR Ca^2+^ gradient [6]. Neuronatin (NNAT) is a small proteolipid (9kD) with high sequence homology to phospholamban [7, 8], which was also recently identified as a novel modifier of estrogen receptor-positive (ER+) breast cancer incidence and survival [9]. Although the role of NNAT in Ca^2+^ homeostasis in breast cancer is unknown, several unrelated studies have collectively implicated elevated NNAT coincided with increased resting level of Ca^2+^ [10, 11], which was attributed to inhibition of SERCA2 activity [12]. Thus, based on these observations [10, 12, 13] and the homology of NNAT with PLN [7, 8], we hypothesized that NNAT potentially modulates intracellular Ca^2+^ levels via interaction with SERCA2. However, it remains yet to be determined whether NNAT modulates other regulators of intracellular Ca^2+^ homeostasis, including store-operated or ion Ca^2+^ channels.

In addition to control of intracellular Ca^2+^ homeostasis, NNAT has been implicated in ROS signaling [14]. Ca^2+^-ROS relationship is a key factor of cell survival during apoptosis and may play a significant importance in cancer cell proliferation and tumorigenesis. For example, it is well established that oxidative stress initiates apoptotic processes through the store-operated channels and mitochondrial-EndoR calcium crosstalk [4, 15, 16]. On the other hand, ROS has been shown to regulate a number of TRPC family members [17]. Peroxisome proliferation is a receptor-mediated process that is activated by the peroxisome proliferator activated receptor (PPAR) and is commonly classified as a tumor promoter by altering gene expression and phenotypically mimicking steroid hormone receptor ligands, such as estrogen [18, 19]. Peroxisome proliferation is produced by H_2_O_2_ and may be one of the main factors mediating an imbalance between the production and degradation of reactive oxygen species in cancer cells. NNAT was recently linked to PPAR levels in adipocytes [20] and was separately colocalized to the peroxisome in the pituitary cells [21]; however, the cancer-specific role of NNAT in peroxisome mediated oxidative stress has not been evaluated.

Here, we provide the first pathophysiological insight into NNAT in Ca^2+^ and ROS signaling in breast cancer. Our data indicate that ROS-mediated NNAT expression strongly regulates cell cycle mechanisms and may control unchecked cell division in cancer. We explore the potential involvement of NNAT in Ca^2+^-ROS relationship as a major mechanism in cell apoptosis and cancer cell survival. Using fluorescent imaging, we reveal the correlation between NNAT expression and Ca^2+^ levels in cytoplasm and EndoR storage. In addition, pharmacological inhibitors of key players in breast cancer cells Ca^2+^ signaling [22], ORAI and TRPC, were used to identify a potential Ca^2+^ pathway in which NNAT modulates Ca^2+^ levels. Collectively, the findings of this study suggest that NNAT couples EndoR Ca^2+^ and ROS signaling with suppression of ER + breast cancer cell proliferation.

## Materials and methods

### Analysis of RNAseq data

RNAseq data from normal and ER+ (N = 699) cases from The Cancer Genome Atlas Breast Invasive Carcinoma (TCGA-BRCA) cohort were downloaded from the Broad GDAC Firehose (https://gdac.broadinstitute.org/). The top correlated genes with NNAT expression were determined by Spearman’s correlation (137 genes correlated; absolute correlation coefficient ≥ 0.5). Enrichment analysis of biological pathways was performed using the Ingenuity Pathway Analysis (IPA) tool. Enrichment analysis of gene ontologies (GO) and network connectivity were performed using the Search Tool for the Retrieval of Interacting Genes (STRING) database (https://string-db.org/).

RNAseq data were assessed for ER + breast cancer cell lines T47D and ZR75 that transgenically expressed NNAT (n = 3) or GFP (n = 3; control). Total RNA was extracted by Trizol followed by library preparation using Illumina’s TruSeq RNA library kit and sequencing on an Illumina HiSeq2500 (Illumina, Inc., San Diego, CA). The Trim Galore program (v0.4.1), only reads with a Phred quality score equal or higher than 20 were taken for analysis. The RSEM program function “rsem-prepare-reference” (v1.3.0) was used to extract the transcript sequences human genome (NCBI Build GRCh38.p2) followed by read alignment using the “rsem-calculate-expression” function. Differential expression analysis was performed using the Bioconductor package DESeq2 version 1.12.4 to compute log2 fold changes and false discovery rate-adjusted p values. Statistical significance of gene expression was determined as FDR < 0.05.

### Cell culture

ER + breast cancer cell lines T47D and ZR75 (luminal B ER + cell lines), MCF10A normal mammary epithelial cells, and MDA-MB-231 triple-negative breast cancer cell lines originally obtained from ATCC (American Type Culture Collection) were cultured in RPMI1640 (Gibco) with 10% FBS. All cell lines were mycoplasma-negative and were cultured for < 6 months.

### NNAT promoter activity

T47D or ZR75 cell lines were seeded in 24-well plates (100k cells/well). 24 h post seeding, each well was transfected using Lipofectamine 2000 or Transit-BrCa with 250 ng of the Dual Luminescence NNAT promoter plasmid (GeneCopoeia) and one of the following plasmids: pLX304 (empty vector control), NRF1, E2F1, E2F4 (250 ng of each), and Peroxisome Proliferator Activated Receptor (PPAR)/Retinoid X Receptor heterodimers: PPARα + RXR, PPARγ + RXR (125 ng of each). 24 h post transfection, media from each well was collected, and luminescence activity was measured based on the manufacturer’s protocol (GeneCopeia).

### NNAT expression during oxidative stress and clofibrate treatment

T47D, ZR75, MCF10A, and MDA-MB-231 cell lines were seeded in 6 well plates at a seeding density of 500k cells/well. 24 h post seeding, each well was treated with or without hydrogen peroxide (H_2_O_2_) (specific concentrations of 100–500 µM were used on the different cell types based on how much H_2_O_2_ induced cell death) for 7 h. Similarly, each well was treated with Clofibrate for 24 h. After that, cells were collected in 1 mL of Trizol, and the total RNA was extracted. The RNA was subsequently made into cDNA for qPCR analysis of CDKN1A, CDKN2B, NNAT, and NRF1 mRNA expression (Supplemental Table 1).

### Characterizing the role of the EndoR localization signal of NNAT protein on ER + breast cancer cell proliferation

The amino acid sequence of NNAT was analyzed through the Eukaryotic Linear Motif (ELM) database, which revealed a consensus EndoR localization sequence (AA75-78). To determine if the EndoR localization signal was required for NNAT function, a lentiviral construct was generated to express NNAT lacking the EndoR sequence (dER). To assess the cell growth effects, 50k cells/well of T47D or ZR75 cell lines were transduced with lentiviral expression constructs expressing the wild-type NNAT or dER NNAT variant in 24-well plates (n = 9 wells per cell line). At 48-hours post seeding, cells were counted with a Countess™ II FL Automated Cell Counter (Life Technologies).

### Characterizing the subcellular localization of NNAT with confocal imaging

Cells were observed under a laser scanning confocal microscope (Nikon A1-R) for dual staining of NNAT and subcellular markers in still images and z-stacks for colocalization. T47D or ZR75 NNAT-GFP tagged cells were seeded on glass bottom culture dishes (20k cells/well; MatTek dishes). At 24 h post seeding, cells were incubated with 2 µl of 1:10,000 dilution of Hoechst 33,342 (ThermoFisher) to stain the nucleus and one of the following organelle markers for the EndoR, ER-Tracker™ Red (Invitrogen), Mitochondria, MitoTracker® Red CMXRos (Invitrogen), Golgi apparatus, CellLight™ Golgi-RFP, BacMam 2.0 (Invitrogen), or Lysosome, Cell Navigator™ Lysosome Staining Kit (ATT Bioquest), in Hank’s Balanced Salt Solution with calcium and magnesium (Gibco) for 30 min.

### Confocal imaging of NNAT-dependent regulation of calcium levels in ER + breast cancer cells

T47D and ZR75 cells overexpressing GFP, NNAT, pLentiCRISPRv2 empty vector control (CRISPR control) or NNAT CRISPR Guide RNA3 (GenScript) (n = 3 plates per group; 20k cells/well) were seeded on glass bottom culture dishes. 24 h after cell seeding, Ca^2+^ imaging was performed with laser scanning confocal microscope (Nikon A1-R). CRISPR knockout was validated via Western blot on NNAT-FLAG cell lines. Basal intracellular calcium [Ca^2+^]_i_ calculations were made based on methods previously described [23]. Briefly, cells were incubated with the fluorescent Ca^2+^ indicator Cal-590™ AM (AAT Bioquest) for 1 h. The cultured media was replaced with a 2 mM Ca^2+^ HEPES buffer solution. Basal fluorescence was recorded for at least 1 min to ensure the signal was stable, at which point 10mM of ionomycin (Sigma) was applied to detect maximum uptake of Ca^2+^. Once the fluorescence reached a max, 10 mM of MnCl_2_ (Sigma) / 2.5 mM of EDTA (AMRESCO) was applied to induce a fluorescent minimum.

To evaluate EndoR Ca^2+^, cells were incubated with Mag-Fluo-4 AM (AAT Bioquest) for 1 h. Media was then replaced with a 0 mM Ca^2+^ HEPES buffer solution. Basal fluorescence was recorded for at least 1 min to ensure the signal was stable, at which point 2 µM of thapsigargin (Sigma) was applied to the cells to induce EndoR Ca^2+^ release. For the inhibition of store-operated calcium entry (SOCE) related calcium channels, cells pretreated with or without 6 μm of pyr6 (ORAI1/3 inhibitor, Sigma) or 5 μm of pyr3 (TRPC3 inhibitor, Sigma) for 5 min. The selectivity between ORAI and TRPC for the pyrazole (pyr) compounds was based on the previous reports [24–26]. The selectivity of the available pharmacological tools for ORAI1 and ORAI3 is limited, and we cannot discriminate between those two CRAC channels known to be expressed in estrogen receptor-positive (ER+) breast cancer cell lines [27, 28]. The area under the curve was analyzed to measure the total amount of Ca^2+^ being released from the EndoR. Images were processed with ImageJ (v.1.51u, NIH).

### Statistical analysis

All statistical analyses were performed using Sigma Plot 11.0 software. Data are presented as ± standard error of the mean (SEM). Data were tested for normality (Shapiro-Wilk) and equal variance (Levene’s homogeneity test). Paired t-test was used to detect the statistical difference between two variables for the same subject. For more than two groups of variables for the same subject, the analysis of variance (ANOVA) was used with corresponding Tukey or Dunnett multiple-comparisons adjustments.

## Results

### NNAT expression correlates with ROS and PPAR signal-transduction pathway in TCGA-BRCA ER + cohort

**Fig. 1:**
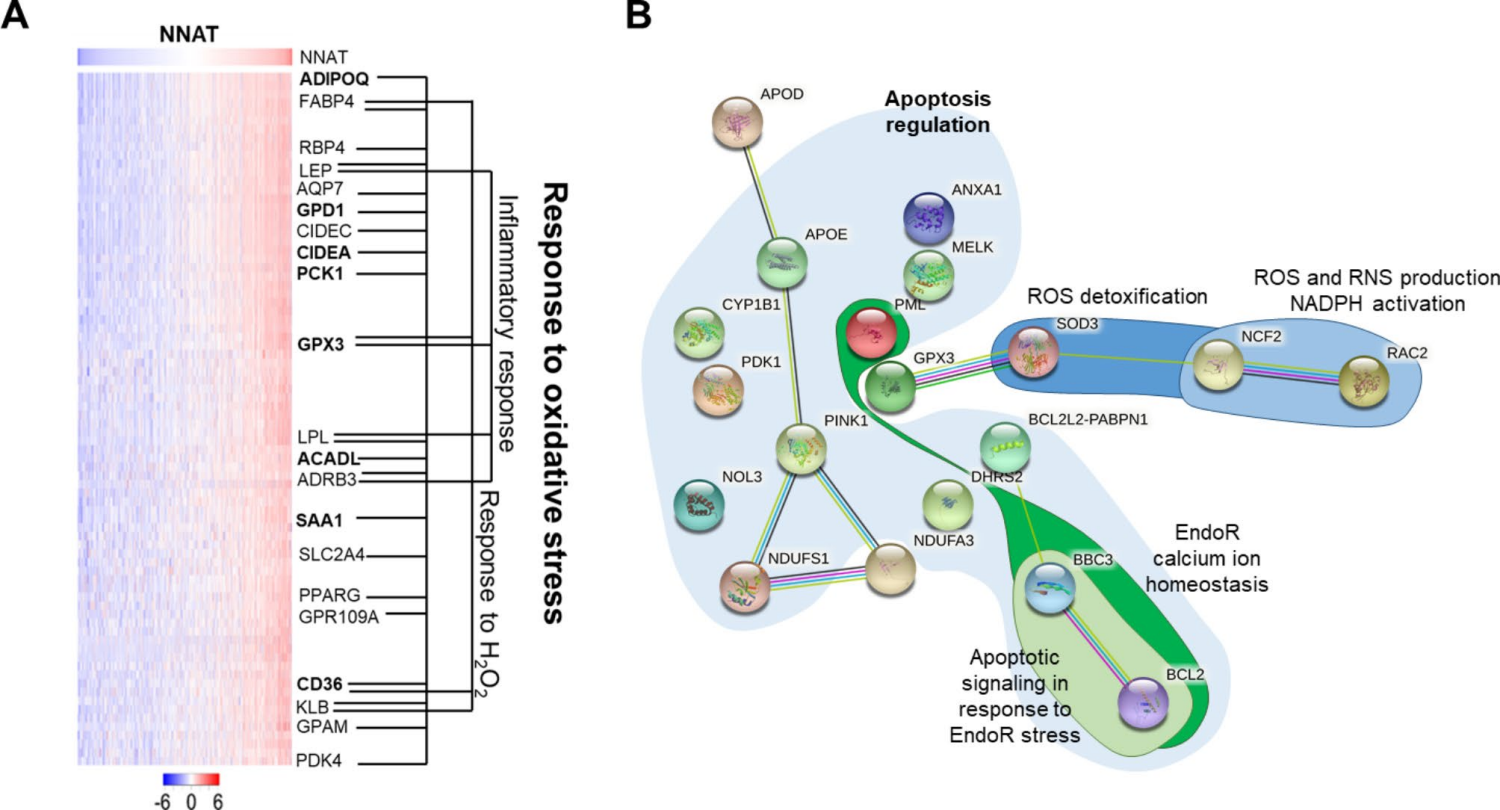
NNAT expression correlated with ROS and PPAR signal-transduction pathway in TCGA-BRCA ER + cohort and ER + breast cancer cells. **A** Gene expression that correlates with NNAT expression in breast cancer patients. Analysis of NNAT mRNA expression in TCGA-BRCA from ER + tumors (n = 699). Data are presented as log expression values and mean values are statistical different between groups (2.2 × 10^− 16^), as tested by ANOVA. **B** The most significant modules of oxidative stress genes selected from protein–protein interaction network upregulated by NNAT overexpression in both ZR75 and T75D cell lines

Elevated NNAT protein in ER + breast tumor biopsies was previously associated with decreased tumorigenic potential and prolonged patient survival [9], yet the underlying regulators of NNAT expression remain elusive. To identify pathways related to NNAT expression, we used publicly available expression data from the ER + breast cancer cohort (N = 699 patients) of TCGA. Ingenuity Pathway Analysis (IPA) analysis of 137 correlated genes (R ≥ 0.5) revealed a significant enrichment (p < 10^− 18^) of ROS and PPAR signaling pathways (Fig. 1A and Supplemental Fig. 1). Likewise, STRING network analysis revealed significant connectivity and enrichment (p < 1.0^− 16^) of ROS and PPAR pathways in TCGA-BRCA ER + patients with elevated NNAT expression (Supplemental Fig. 2). Finally, STRING analysis of transcriptomics data following exogenous overexpression of NNAT in ER + breast cancer cell models, T47D and ZR75, revealed enrichment of oxidative stress and apoptosis pathways (Fig. 1B; Table 1), in addition to the inhibition of cell cycle pathways reported Plasterer et al. [9]. Combined with our previous observations that NNAT suppresses ER + breast cancer cell proliferation [9], these data collectively suggested that NNAT integrates cell cycling with oxidative stress and peroxisome proliferation signaling.

**Table 1:** **Reactive oxygen and calcium related genes behavior comparison between NNAT overexpression ER + breast cancer cells lines (T47D and ZR75) vs. poor prognosis tumorigenesis in clinical reports.** Opposite expression patterns marked in bold

NNAT overexpression significantly regulated genes	During NNAT overexpression	Expression pattern under tumorigenesis and/or poor prognosis
**PML**	↑	↓ [61] breast cancer
**MELK**	↓	↑ [62] breast cancer
**NOL3**	↑	↓ [63] myeloid malignancies
APOE	↑	↑ [64] melanoma and glioblastoma exhibited accelerated tumor growth in ApoE-deficient mice
**SOD3**	↑	↓ [65] breast cancer, head and neck cancer, lung cancer, and sarcoma
**PINK1**	↑	↓ [66] breast, colorectal, esophageal, head and neck, liver and ovarian cancers, leukemia and melanoma
**NDUFS8**	↑	↓ [67] Renal cell carcinoma
**NDUFS1**	↓	↑ [68] breast cancer (The Human Protein Atlas data)
NDUFA3	↑	↓↑ [68] breast cancer (The Human Protein Atlas data)
**CYP1B1**	↓	↑ [69] breast, brain, colon, ovarian, and prostate cancers
GPX3	↓↑	↓ [70] breast cancer
**PDK1**	↓	↑ [71] breast cancer
**DHRS2**	↓	↓ [72] breast cancer
**ANXA1**	↓	↑ [73] breast cancer
**APOD**	↓	↑ [74] breast cancer
**NCF2**	↓	↑ [75] kidney Renal Clear Cell Carcinoma
**RAC2**	↑	↓ [68] breast cancer (The Human Protein Atlas data)
BCL2	↓	↓ [76] breast cancer
BCL2L2-PABPN1	↑	↑ [77, 78] glioblastoma
**BBC3**	↑	↓ [79] head and neck cancer

### NNAT expression is regulated by ROS and PPAR signaling that coincides with decreased breast cancer proliferation

**Fig. 2:**
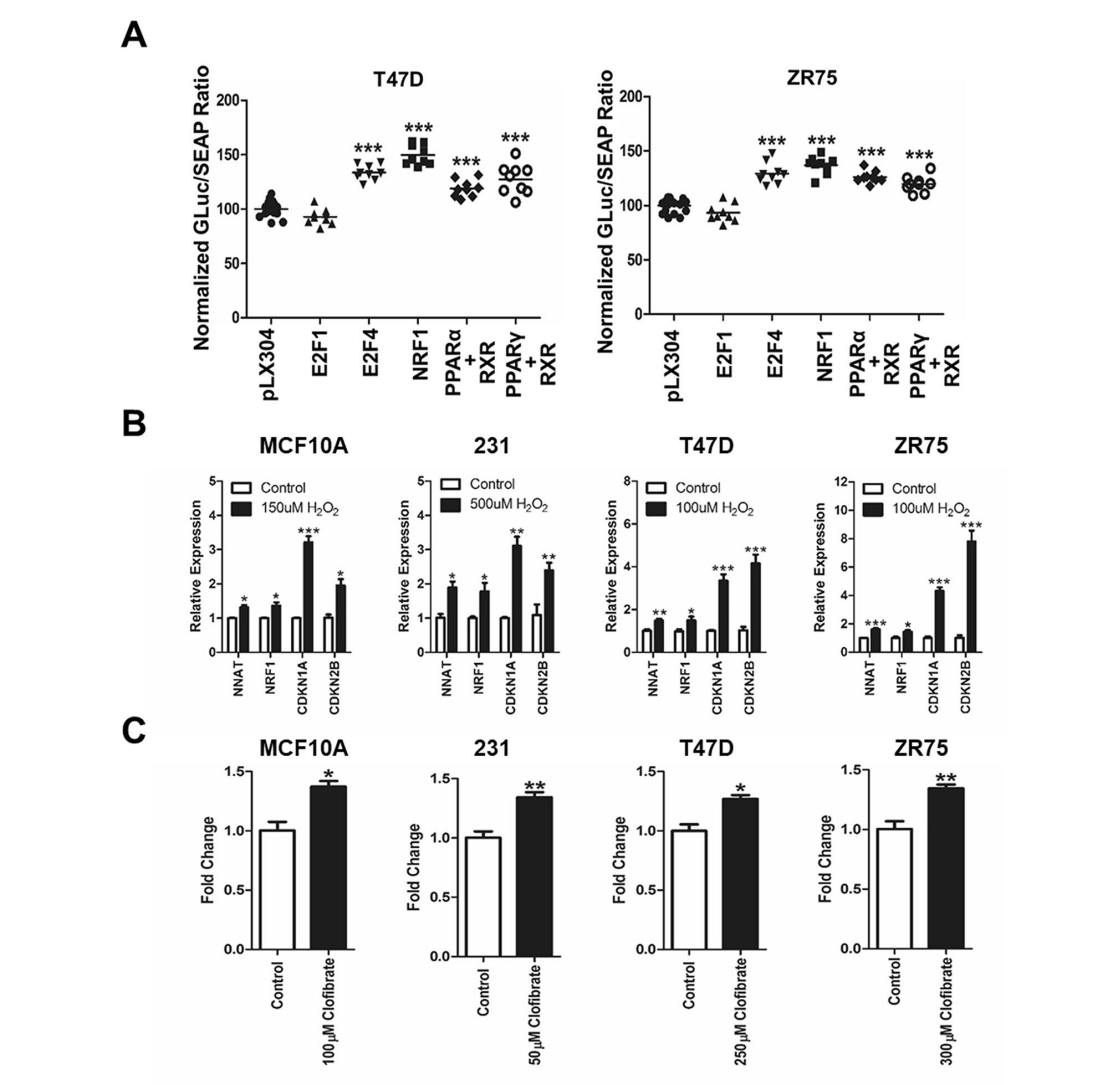
NNAT mRNA expression is regulated by oxidative stress and activation of PPAR signaling cascade. **A** Luminescence activity of NNAT promoter activity co-transfected with E2F1, E2F4, NRF1, PPARα + RXR, PPARγ + RXR, or pLX304 control plasmid in ER + breast cancer cell lines. Data presented as mean percentage of GLuc/SEAP ratio normalized to pLX304 ± SEM (n = 9 per group; paired t-test, ***p < 0.001 vs. pLX304 control). **B** Evaluation of mRNA expression of NNAT, tumor suppressors genes CDKN1A and CDKN2B, and the transcription factor NRF1 responsible for cellular growth, during the exposure to H_2_O_2_. MCF10A, MDA-MB-231, T47D, and ZR75 cell lines untreated or treated with H_2_O_2_ (n = 3 per group; paired t-test, *p < 0.05, **p < 0.01, ***p < 0.001 vs. control). **C** NNAT mRNA expression of MCF10A, MDA-MB-231, T47D, and ZR75 cells treated with or without PPAR agonist, Clofibrate (n = 3 per group; paired t-test, *p < 0.05, **p < 0.01 vs. untreated control)

In silico analysis of the NNAT promoter revealed consensus binding sites for transcriptional regulators of the cell cycle (E2F1, E2F4), oxidative stress (NRF1), and peroxisome proliferation (PPAR), prompting further investigation of transcriptional regulation of NNAT by dual-luciferase promoter assay. Briefly, the ER + breast cancer cell lines, T47D and ZR75, were co-transfected with the NNAT promoter-reporter construct, and plasmids that constitutively expressed E2F1, E2F4, NRF1, PPARα/RXR, PPARγ/RXR, or control (pLX304). At 24 h post-transfection, NNAT promoter activity was significantly elevated by E2F4, NRF1, PPARα/RXR, and PPARγ/RXR compared to the empty vector control (p < 0.001), whereas E2F1 showed no effects (Fig. 2A). Likewise, NNAT expression was significantly elevated in MCF10A, MDA-MB-231, T47D, and ZR75 cells treated with the PPAR agonist, clofibrate, or under oxidative stress conditions (H_2_O_2_ 100 μm), which also coincided with the elevation of the cell cycle inhibitors, CDKN1A and CDKN2B (Fig. 2B-C). Collectively, these data confirmed that NNAT is transcriptionally elevated in ER + breast cancer cells undergoing oxidative stress and peroxisome proliferation.

### NNAT colocalizes to the endoplasmic reticulum and lysosome in ER + breast cancer

**Fig. 3:**
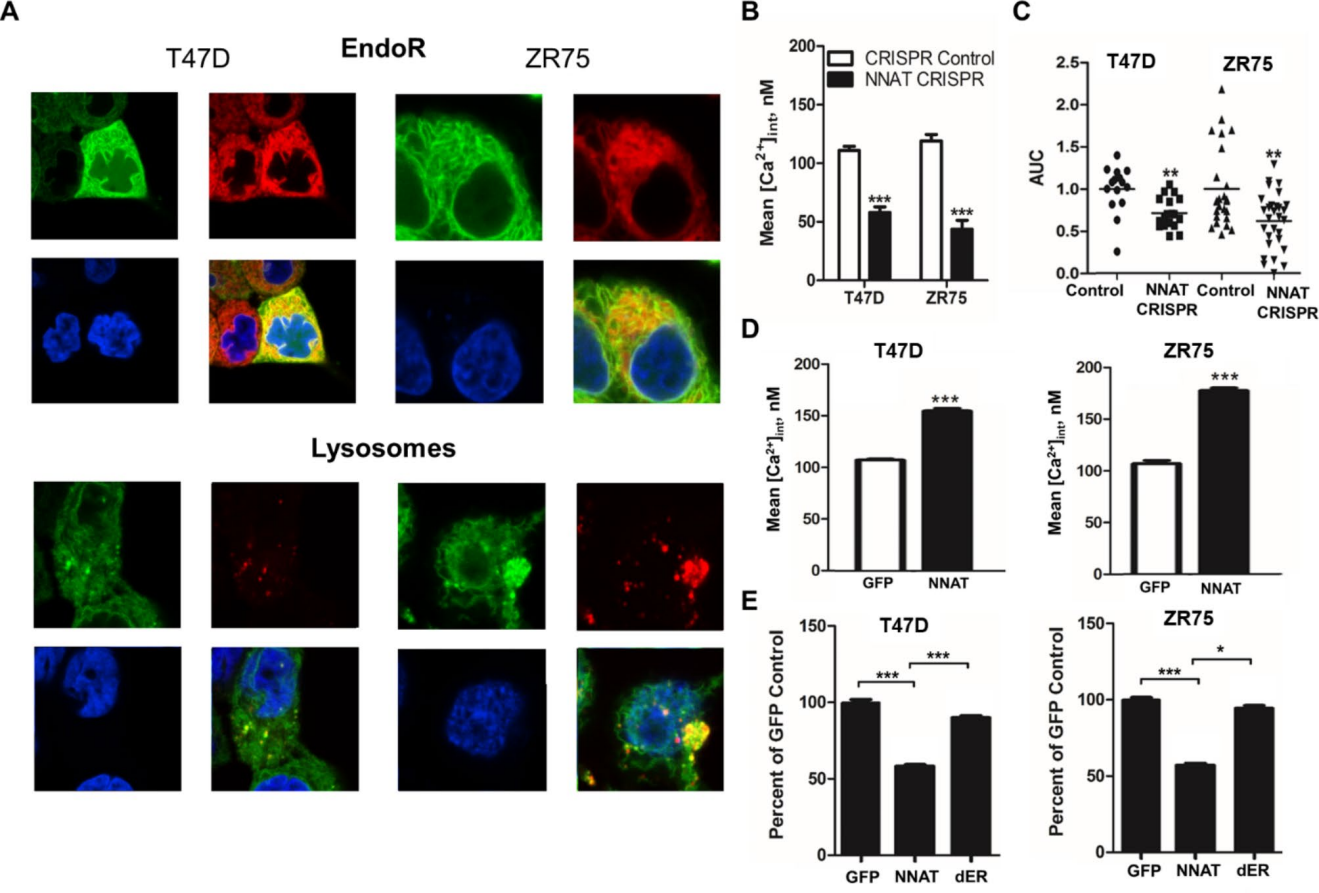
NNAT regulates intracellular calcium in ER + breast cancer cells through EndoR calcium storage. **A** Confocal fluorescent imaging revealed NNAT (green) colocalization with EndoR and lysosome (red and yellow merged images). **B** Basal Ca^2+^ concentration in T47D and ZR75 CRISPR knockout of NNAT cell lines (NNAT CRISPR) (n = 30 per group, paired t-test, p < 0.001 vs. control). **C** EndoR Ca^2+^ release following knockout of NNAT (n ≥ 16 per group; paired t-test, **p < 0.01 vs. respective CRISPR control). **D** Basal Ca^2+^ concentration in T47D and ZR75 cell lines overexpressing NNAT (n = 30 per group, paired t-test, p < 0.001 vs. control). **E** Change in proliferative capacity in T47D and ZR75 breast cancer cell lines overexpressed NNAT deletion construct (wild-type, NNAT, and dER). (n = 9 per group; one-way ANOVA tests (Tukey post hoc test) *p < 0.05, ***p < 0.001 vs. GFP).

Prediction of NNAT functional sites using the Eukaryotic Linear Motif database (http://elm.eu.org/search.html) revealed a transmembrane domain and an endoplasmic reticulum (EndoR) retention sequence, suggesting that NNAT is localized to the EndoR of breast cancer cells. To test this hypothesis, the subcellular localization of NNAT-GFP in T47D and ZR75 cells was assessed by fluorescent confocal imaging using organelle-specific dyes for the EndoR, lysosome, Golgi apparatus, and mitochondria. As shown in Fig. 3A, NNAT protein (green) was largely colocalized with the EndoR and lysosome (yellow/merged images) in both T47D and ZR75 cell lines. In contrast, NNAT expression was not colocalized with markers for the Golgi apparatus or mitochondria (Supplemental Fig. 3). Based on the observation that NNAT expression correlated with oxidative stress pathways in ER + breast cancer patients, these data suggest that NNAT might mediate EndoR function(s) in the cellular response to ROS that impacts the progression of ER + breast cancer.

### NNAT regulates intracellular calcium in ER + breast cancer cells through EndoR calcium storage

Intracellular Ca^2+^ homeostasis is altered in cancer cells and involved in tumor initiation, angiogenesis, progression and metastasis [29]. To assess the impact of NNAT on Ca^2+^ homeostasis in breast cancer cells, intracellular free Ca^2+^ levels detected by Cal-590™ AM staining was measured by confocal microscopy in T47D and ZR75 cells lacking NNAT expression (Supplemental Fig. 4). Compared with non-targeting controls, depletion of endogenous NNAT by CRISPR resulted in a significant decrease in intracellular free Ca^2+^ levels (Fig. 3B). Likewise, the loss of NNAT significantly decreased Ca^2+^ in the cytoplasm and EndoR, as estimated by the presence of the SERCA inhibitor, thapsigargin (Fig. 3C). In comparison, stable overexpression of NNAT in T47D and ZR75 cells significantly increased basal cytoplasmic Ca^2+^ concentration (Fig. 3D), which coincided with significantly decreased tumor cell proliferation compared with control cells (Fig. 3E). Notably, proliferation of T47D and ZR75 cells was not suppressed by overexpression of NNAT lacking the putative EndoR retention signal (Fig. 3E and Supplemental Fig. 4), suggesting that the tumor suppressive role(s) of NNAT function through the EndoR. Collectively, these data, combined with our previous observations that elevated NNAT correlated with better survival of ER + breast cancer patients [9], suggest that NNAT is a key regulator of Ca^2+^ homeostasis that impacts ER + breast cancer cell progression.

### ORAI but not TRPC3 inhibition reduces NNAT-mediated elevation in intracellular Ca^2+^ concentration in breast cancer cells.

**Fig. 4:**
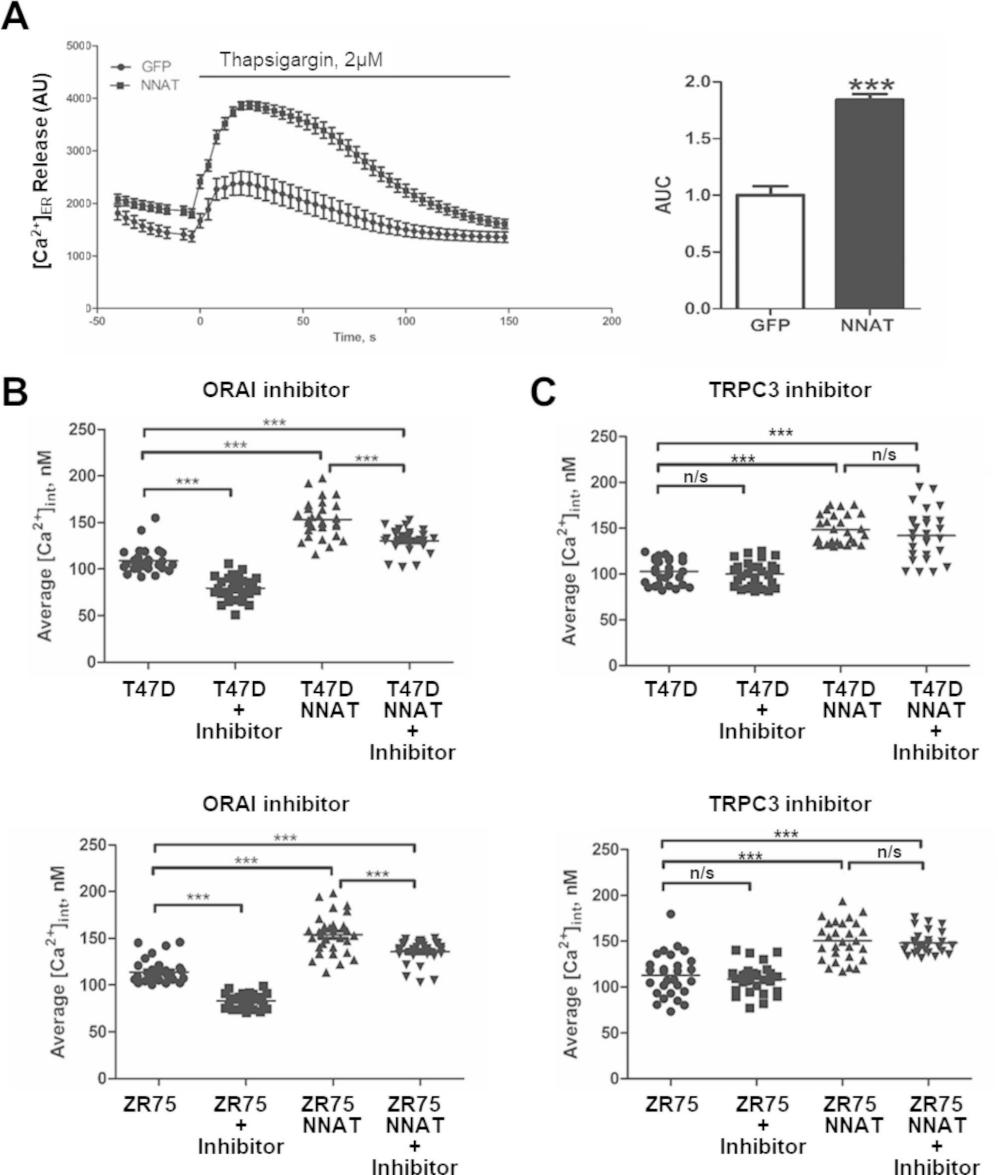
ORAI but not TRPC3 inhibition reduces NNAT-mediated elevation in intracellular Ca^2+^concentration in breast cancer cells. The overexpression of NNAT promotes significantly higher Ca^2+^ release from EndoR of ZR75 breast cancer cells. (n = 30 per group; paired t-test, ***p < 0.001 vs. control GFP) (**A**). Intracellular Ca^2+^ levels in standard and overexpressing NNAT T47D and ZR75 cell lines in the presence or absence of ORAI (pyr6) (**B**) or TRPC3 (pyr3) (**C**) pyrazole compound inhibitors (n = 30 per group; Two-way ANOVA tests (factors: NNAT and inhibitor; Tukey post hoc test) ***p < 0.001)

Intracellular Ca^2+^ levels are restored by (SOCE) upon EndoR depletion, and recent studies have shown that SOCE function is required for ER + breast cancer growth and progression [30]. SOCE is mediated via STIM1 and native pore-forming unit ORAI, and we confirmed the presence of both ORAI1 and ORAI3 in ZR751 and T47D lines ( [27, 28].; 10.6084/m9.figshare.22779170.v1). To test the role of SOCE in NNAT-mediated regulation of intracellular Ca^2+^ levels, ER + breast cancer cells overexpressing NNAT or empty vector control were treated with selective blockers of ORAI (pyr6) or TRPC (pyr3) channels, followed by measurement of intracellular Ca^2+^ by fluorescent confocal microscopy. The NNAT overexpression significantly increases Ca^2+^ release from the EndoR estimated by thapsigargin application (Fig. 4A). Compared with the empty vector controls, NNAT overexpression significantly elevated intracellular Ca^2+^ levels in both ER + breast cancer cell lines, which was reduced by blockade of ORAI channels (Fig. 4B) but not TRPC channels (Fig. 4C). Compared with the empty vector controls, NNAT overexpression significantly elevated intracellular Ca^2+^ levels in both ER + breast cancer cell lines, which was reduced by blockade of ORAI channels (Fig. 4B) but not TRPC channels (Fig. 4C). Notably, although inhibition of ORAI channels significantly reduced intracellular Ca^2+^ in the presence of NNAT overexpression, this reduction did not approach the level of ORAI channel blockade in the parental cells (Fig. 4B). Collectively, these data suggest that NNAT modulates homeostatic levels of intracellular Ca^2+^ by shifting the equilibrium of Ca^2+^ at steady state between intracellular stores and the extracellular space.

## Discussion

We previously demonstrated that NNAT suppresses cell proliferation and migration, which correlates with decreased tumorigenic potential and prolonged survival in ER + breast cancer patients [9]. Elsewhere, NNAT has been reported to function as an intracellular Ca^2+^ regulator [10, 23, 31], leading to the hypothesis that NNAT suppresses ER + breast cancer by altering Ca^2+^ homeostasis in response to extracellular stimuli, such as ROS. Further, ROS and Ca^2^ homeostasis have long been implicated in tumorigenesis and progression across many cancers [4], yet the underlying mechanisms remain incompletely understood. To our knowledge, this study was the first to provide a mechanistic link between ROS and elevated NNAT expression and the downstream effects of NNAT on the regulation of intracellular Ca^2+^ through the EndoR that coincided with decreased proliferation of ER + breast cancer cells.

### NNAT is elevated in ER + breast cancer by ROS and PPAR signaling

The role of ROS in breast cancer is based on intracellular levels; at low levels, ROS can promote proliferation in cancer by stimulating cyclin D1 expression, triggering ERK phosphorylation and MAPK activation, which are all associated with carcinogenesis and cancer cell survival [32–35]. The accumulation of ROS may induce irreparable damages subsequently resulting in tumor cell apoptosis and is used in some cancer therapy strategies [36]. ER + breast cancer patient RNAseq data from TCGA revealed an association of ROS and PPAR signaling with NNAT expression and *in silico* analysis of the NNAT promotor revealed consensus binding sites for NRF1 and PPARα/PPARγ, as well as the cell cycle transcription factor, E2F4. Co-transfection of the NNAT promoter-reporter constructs with vectors expressing NRF1, PPARα, PPARγ, and E2F4 increased NNAT promoter activity, suggesting that NNAT expression is transcriptionally regulated ROS and PPAR signaling in the context of cell cycling.

Exposure of ER + breast cancer cells to the ROS inducer, H_2_O_2_, or PPAR agonist, clofibrate, increased NNAT abundance, confirming that ROS and PPAR signaling activate NNAT expression. PPAR signaling is also known to elevate ROS production and decrease cancer progression [37–40], suggesting that NNAT expression might integrate ER + breast cancer response to both stimuli with cell cycling. Further supporting this hypothesis was the observation that NNAT elevation by ROS coincided with the upregulation of the cell cycle inhibitors, CDKN1A and CDKN2B. Additionally, E2F4 is a negative regulator of the cell cycle by increasing the expression of genes that inhibit the cell cycle [41]. Collectively, the observations that NNAT is transcriptionally regulated by E2F4 and that overexpression of NNAT decreased ER + breast cancer cell proliferation suggest that cell cycle inhibition in response to ROS or PPAR signaling is potentially sensed and reinforced by NNAT. This hypothesis also fits with our previous observations that NNAT overexpression decreased the expression of genes related to cell cycle progression and activated genes related to cell cycle inhibition [9]. Increasing ROS levels has been shown to directly trigger the release of lysosomal calcium, leading to lysosomal autophagy [42]. NNAT is localized in lysosome and its expression is regulated by ROS production. These data combined lead to the hypothesis that NNAT may be a mediator of lysosomal autophagy through increased ROS production.

### Role of NNAT in regulating Ca^2+^ homeostasis in ER + breast cancer cells

Confocal fluorescent microscopy revealed that NNAT co-localized to the EndoR and modulation of NNAT expression modify intracellular Ca^2+^ homeostasis in ER + breast cancer cells, fitting with reports that NNAT regulates intracellular Ca^2+^ in other physiological and pathophysiological settings [10, 31]. NNAT has been hypothesized to regulate SERCA2 on the EndoR membrane based on homology to phospholamban and sarcolipin [7, 43]. SERCA2 is important regulator of a EndoR Ca^2+^ during apoptosis and integral in maintaining Ca^2+^ levels to support the protein synthesis and folding machinery of the ER [44, 45]. Cancer cells were shown to inhibit Ca^2+^-ROS dependent apoptosis and inactivation of SERCA may be one of the mechanisms underlying this process [46]. To test the relevance of NNAT and EndoR Ca^2+^ storage, the SERCA2 inhibitor, thapsigargin [47], was used to induce EndoR Ca^2+^ release into the cytosol and indirectly measure the total amount of Ca^2+^ in the EndoR, which revealed that NNAT levels correspond with changes in EndoR Ca^2+^ storage. Combined with the observations that NNAT colocalized to the EndoR and required the EndoR localization sequence for antiproliferative effects, the data collectively suggest that NNAT suppresses ER + breast cancer by altering EndoR Ca^2+^ storage.

The SOCE channels are required to replenish EndoR Ca^2+^ levels after store depletion [48], which is mediate by activation of STIM1 proteins in the EndoR membrane that translocate to the cell membrane and activate channels comprised of either ORAI or TRPC proteins [49–54]. ORAI channels are a well-characterized regulators of the proliferation and migration of many basal breast cancer cells [28]. ORAI1 is responsible SOCE-regulated calcium entry in a variety of cell types, and ER + breast cancer cells used in this study were reported to show normal or decreased ORAI1 expression [27]. In addition, recent data suggest that ORAI3 plays crucial role in calcium regulation of ER + breast cancer cells in particular [27, 28], and is capable of complexing with ORAI1 to form heteromultimeric channel [55]. The differential expression of ORAI isoforms directly related to the redox sensitivity, since ORAI3 is lacking redox sensor [56]. TRPC channels are known to be activated by ROS [57], and TRPC3 was proposed to interact with STIM1/ORAI1 complexes promoting Ca^2+^ entry [58]. We hypothesize that NNAT might increase intracellular Ca^2+^ levels and EndoR Ca^2+^ storage by activating SOCE function. To test this hypothesis, we used specific inhibitors of ORAI or TRPC channels, of which only ORAI inhibition modulate Ca^2+^ homeostasis. Notably, ORAI channels have been shown to mediate migration, metastasis, and proliferation in breast cancer cells [59, 60], suggesting that SOCE is critical to breast cancer progression. Overall, we propose that ROS activate NNAT expression, and high levels of NNAT regulates equilibrium of EndoR Ca^2+^ through SERCA function, which shifts the steady state between intracellular stores and the extracellular space.

## Conclusions

Collectively, our data suggest that NNAT expression is associated with ROS and PPAR signaling in ER + breast cancer patients from the TCGA cohort. NNAT was localized in the endoplasmic reticulum and lysosomes of ER + breast cancer cells. Overexpression of NNAT in ER + breast cancer cell lines suppresses proliferation and leads to elevated basal calcium levels and calcium release induced by thapsigargin, while NNAT CRISPRKO reduces intracellular calcium levels. NNAT increases intracellular Ca^2+^ levels and EndoR Ca^2+^ storage by activating SOCE function through ORAI channels.

## Electronic supplementary material

Below is the link to the electronic supplementary material.

Supplementary Material 1 - Supplemental Table 1 qPCR Primers.

Supplementary Material 2 - Supplemental Figure 1. NNAT expression and genetic correlation in ER+ breast cancer TGCA RNAseq data.

Supplementary Material 3 - Supplemental Figure 2. STRING analyses in TCGA-BRCA ER+ cohort.

Supplementary Material 4 - Supplemental Figure 3. Confocal imaging for NNAT colocalization with Golgi apparatus and mitochondria.

Supplementary Material 5 - Supplemental Figure 4. Western blotting confirmation of NNAT CRISPR knockout.

Supplementary Material 6 - Supplemental Figure 5. NNAT protein structure with predicted functional motifs.

## Data Availability

The data during the current study are available from the corresponding author on reasonable request.

## Abbreviations

[Ca^2+^]_i_: Intracellular calcium
APC/C: Motif anaphase- promoting complex or cyclosome
E2F1: Transcription factor E2F1
EndoR: Endoplasmic reticulum
ER+: Estrogen receptor
GFP: Green fluorescent protein
GO: Gene Ontology
NNAT: Neuronatin
NRF1: Nuclear respiratory factor 1
ORAI1: Calcium release-activated calcium channel protein 1
PPARγ: Peroxisome proliferator-activated receptor gamma type
ROS: Reactive oxygen species
SERCA2: Sarco/endoplasmic reticulum Ca^2+^-ATPase
SOCE: Store-operated calcium entry
STAT5: Signal transducer and activator of transcription 5
STIM1: Stromal interaction molecule 1
STRING: Search Tool for the Retrieval of Interacting Genes
TCGA-BRCA: The Cancer Genome Atlas Breast Invasive Carcinoma
TCGA: The Cancer Genome Atlas
TRPC3: Short transient receptor potential channel 3
