# Supplementary material for: NNAT is a novel mediator of oxidative stress that suppresses ER + breast cancer
Source: Mol Med. 2023 Jul 3;29:87. doi: 10.1186/s10020-023-00673-y (PMC10318825; doi:10.1186/s10020-023-00673-y)
Supplement: Supplementary file 1 — Supplementary Material 1 - Supplemental Table 1 qPCR Primers. [file 10020_2023_673_MOESM1_ESM.docx]

Supplemental Table 1 qPCR Primers

| Gene | Forward Primer | Reverse Primer |
| --- | --- | --- |
| NRF1 | ATGTCCGCACAGAAGAGCAA | TTCCCGCCCATGCTGTTTAT |
| NNAT | TCATCATCGGCTGGTACATC | CTGTGTCCCTGGAGGATTTC |
| CDKN1A | TGTCCGTCAGAACCCATGC | AAAGTCGAAGTTCCATCGCTC |
| CDKN2B | TATCTGGCCCTCGACACTCA | CACTGCCTTCTCCCACTCAG |
