# Supplementary material for: NNAT is a novel mediator of oxidative stress that suppresses ER + breast cancer
Source: Mol Med. 2023 Jul 3;29:87. doi: 10.1186/s10020-023-00673-y (PMC10318825; doi:10.1186/s10020-023-00673-y)

**Supplemental Figure 1.** Functional relationships between NNAT expression and 137 identified genes indicates a genetic correlation in ER+ breast cancer TGCA RNAseq data (absolute correlation coefficient ≥0.5; N=699). Using the Ingenuity Pathway Analysis (IPA) system Reactive Oxygen Species (ROS) and PPAR signaling pathways were found to be enriched (p<10-18).


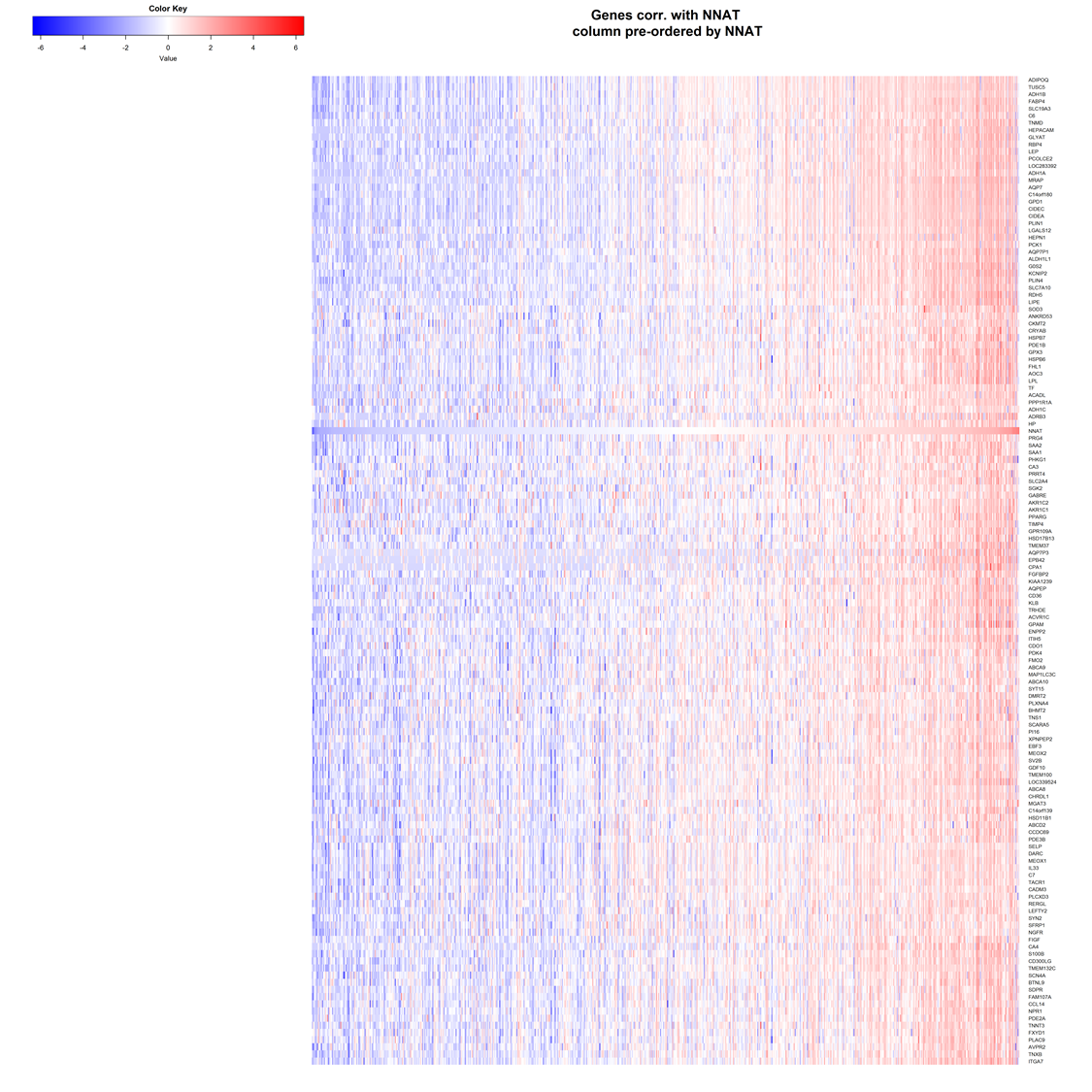

Supplement: Supplementary file 2 — Supplementary Material 2 - Supplemental Figure 1. NNAT expression and genetic correlation in ER+ breast cancer TGCA RNAseq data. [file 10020_2023_673_MOESM2_ESM.docx]
