# Supplementary material for: NNAT is a novel mediator of oxidative stress that suppresses ER + breast cancer
Source: Mol Med. 2023 Jul 3;29:87. doi: 10.1186/s10020-023-00673-y (PMC10318825; doi:10.1186/s10020-023-00673-y)

**Supplemental Figure 2.**

STRING analyses in TCGA-BRCA ER+ cohort indicate that NNAT-dependent ROS related genes mediate elevation in peroxisome proliferator–response elements and PPAR signal-transduction pathway.


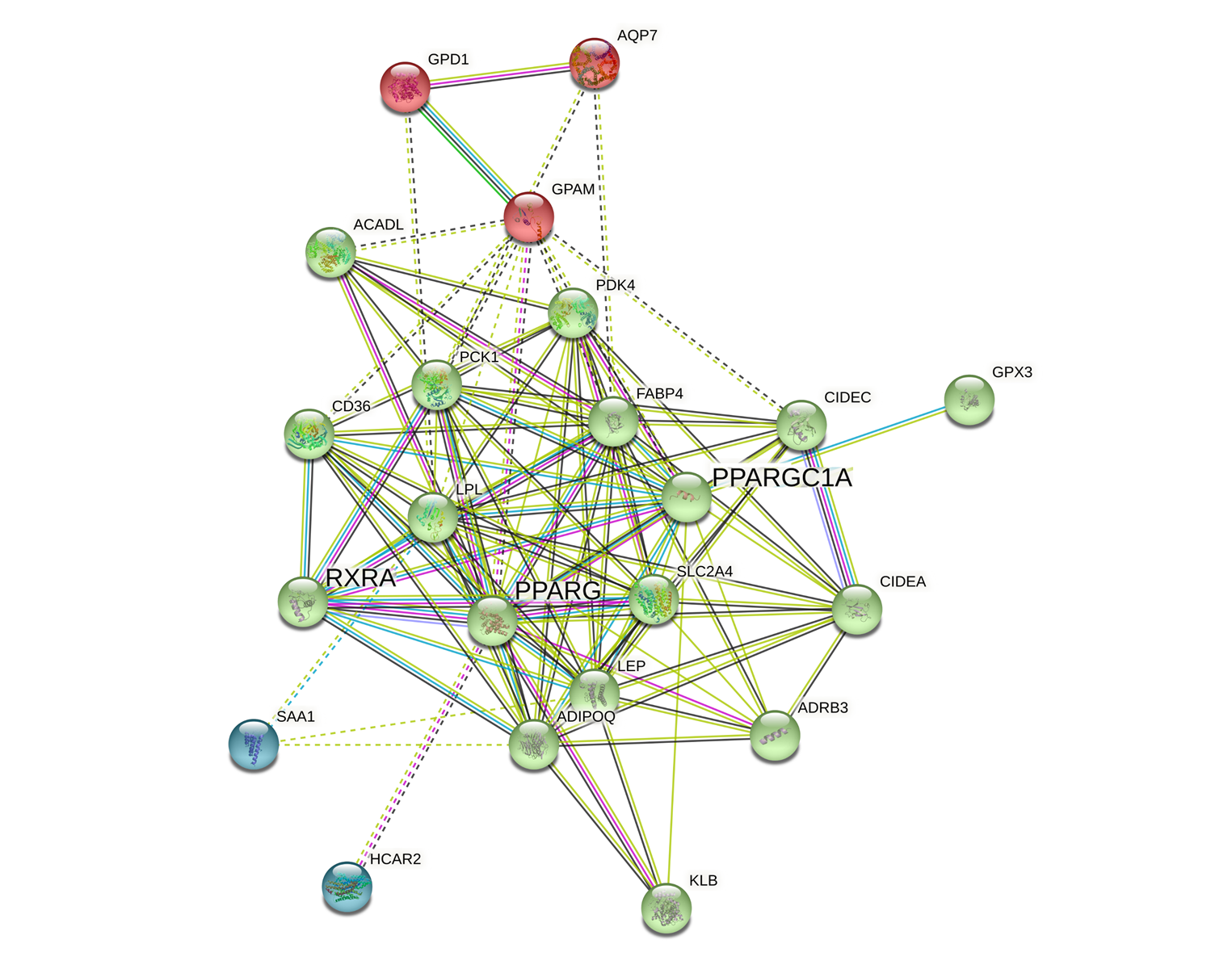

Supplement: Supplementary file 3 — Supplementary Material 3 - Supplemental Figure 2. STRING analyses in TCGA-BRCA ER+ cohort. [file 10020_2023_673_MOESM3_ESM.docx]
