# Supplementary material for: NNAT is a novel mediator of oxidative stress that suppresses ER + breast cancer
Source: Mol Med. 2023 Jul 3;29:87. doi: 10.1186/s10020-023-00673-y (PMC10318825; doi:10.1186/s10020-023-00673-y)

**Supplemental Figure 3.** NNAT expression was not colocalized with markers for the Golgi apparatus or mitochondria. Confocal fluorescent imaging for NNAT (green) colocalization with Golgi apparatus or mitochondria (red).


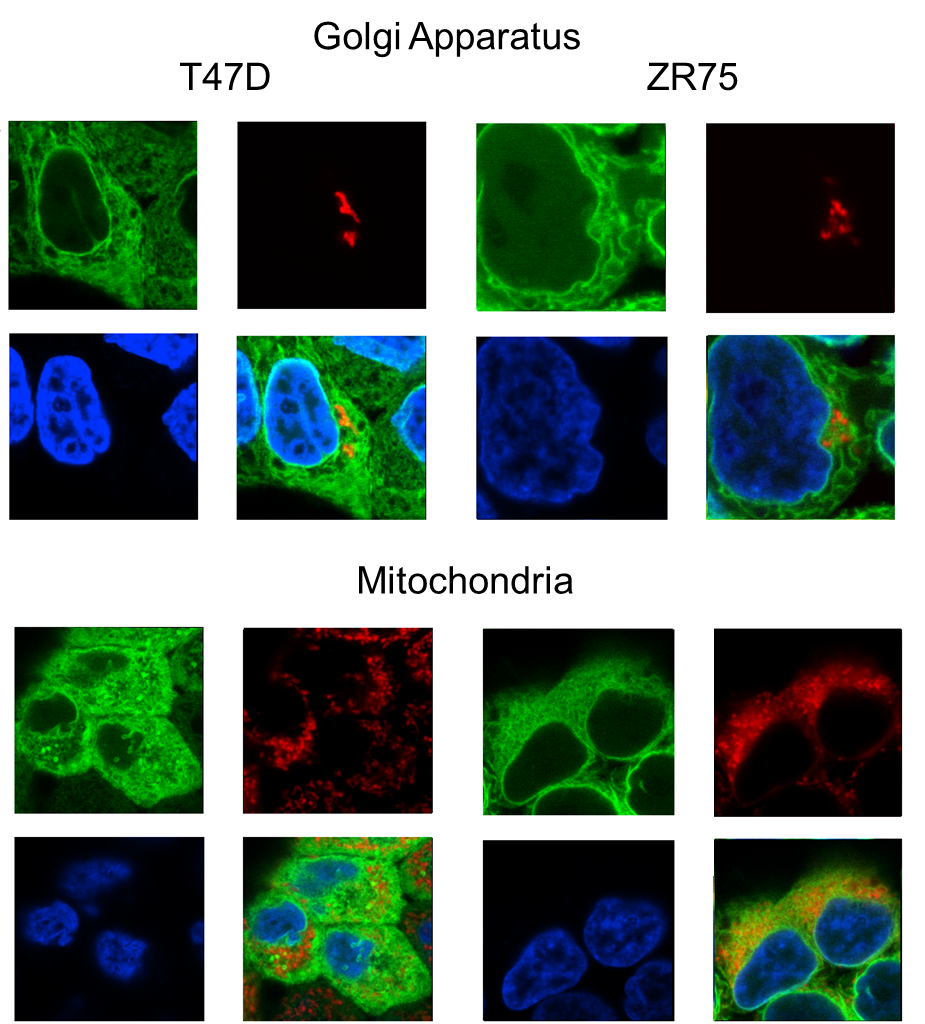

Supplement: Supplementary file 4 — Supplementary Material 4 - Supplemental Figure 3. Confocal imaging for NNAT colocalization with Golgi apparatus and mitochondria. [file 10020_2023_673_MOESM4_ESM.docx]
