# Supplementary material for: NNAT is a novel mediator of oxidative stress that suppresses ER + breast cancer
Source: Mol Med. 2023 Jul 3;29:87. doi: 10.1186/s10020-023-00673-y (PMC10318825; doi:10.1186/s10020-023-00673-y)

**Supplemental Figure 4.** Western blotting confirmed the successful CRISPR knockout of NNAT (NNAT CRISPR) in both T47D and ZR75 cell lines.


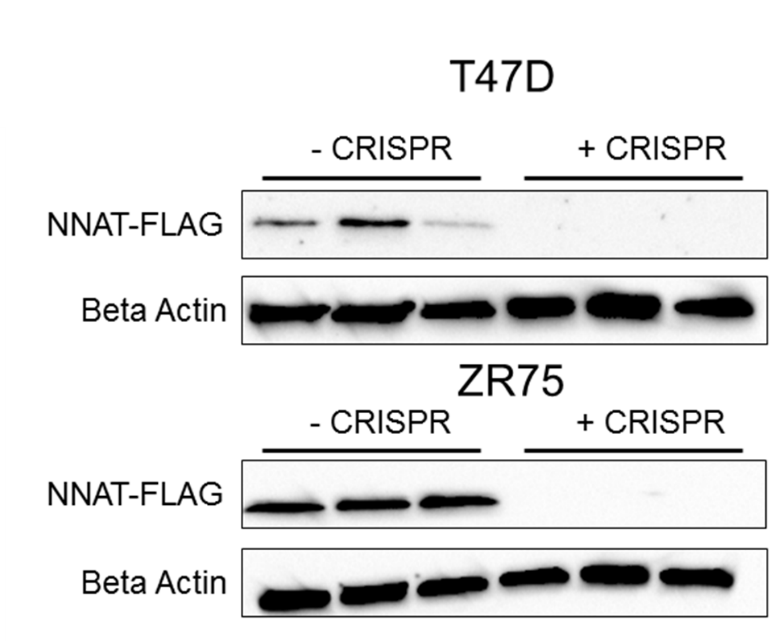


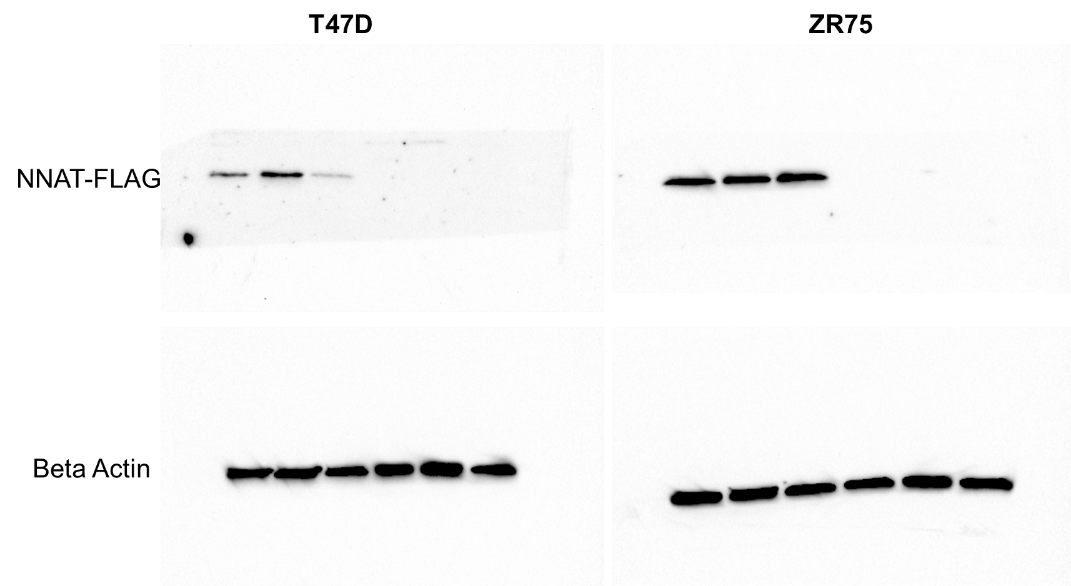

Supplement: Supplementary file 5 — Supplementary Material 5 - Supplemental Figure 4. Western blotting confirmation of NNAT CRISPR knockout. [file 10020_2023_673_MOESM5_ESM.docx]
