# Supplementary material for: NNAT is a novel mediator of oxidative stress that suppresses ER + breast cancer
Source: Mol Med. 2023 Jul 3;29:87. doi: 10.1186/s10020-023-00673-y (PMC10318825; doi:10.1186/s10020-023-00673-y)

**Supplemental Figure 5.** NNAT protein structure with predicted functional motifs. NNAT protein sequence was analyzed for predicted protein motifs using the Eukaryotic Linear Motif resource (http://elm.eu.org/). Based on this analysis, EndoR predicted motifs of interest were identified (ER) and deletion constructs generated with corresponding amino acid sequence which was deleted.


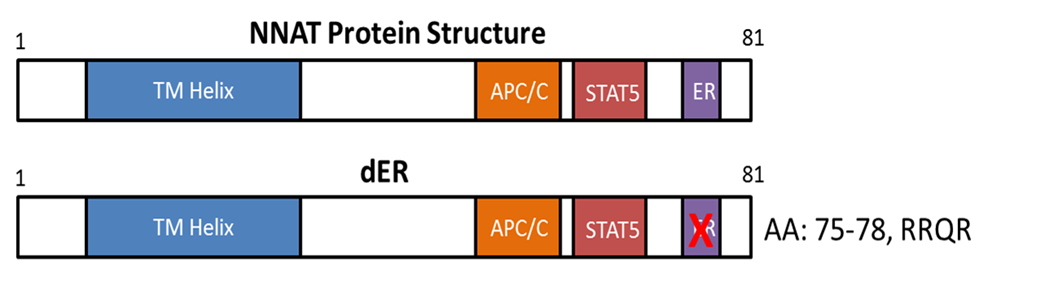

Supplement: Supplementary file 6 — Supplementary Material 6 - Supplemental Figure 5. NNAT protein structure with predicted functional motifs. [file 10020_2023_673_MOESM6_ESM.docx]
